# Editorial: Cell biology of microglia

**DOI:** 10.3389/fncel.2023.1210124

**Published:** 2023-05-19

**Authors:** M. Rosario Sepúlveda, João B. Relvas, Francesca Peri, Veronika E. Neubrand

**Affiliations:** ^1^Department of Cell Biology, Faculty of Sciences, University of Granada, Granada, Spain; ^2^Instituto de Investigação e Inovação em Saúde and Instituto de Biologia Molecular e Celular (IBMC), University of Porto, Porto, Portugal; ^3^Department of Biomedicine, Faculty of Medicine, University of Porto, Porto, Portugal; ^4^Department of Molecular Life Sciences, University of Zurich, Zurich, Switzerland

**Keywords:** microglia, Nerve Growth Factor (NGF), Apolipoprotein D (apoD), immune priming, toll-like receptor (TLR), lipopolysaccharide (LPS), retina, extracellular vesicles

Microglia are the resident immune cells of the Central Nervous System (CNS) and play a key role during development, synapse formation, CNS plasticity and defense across life (Wolf et al., 2017). In the adult and aging brain, microglia have been identified as important players in the progression of neurodegenerative diseases with an underlying neuroinflammation. Hence, microglia are responsive to a variety of stimuli and, correspondingly, can acquire many different phenotypes (Paolicelli et al., 2022). These range from neuroprotective phenotypes, supporting the CNS, to neuroinflammatory phenotypes with fatal consequences for neurons and their environment. Although the existence of microglia has been known for more than 100 years, some of their essential functions were revealed only in recent decades. Furthermore, with technical advances in sequencing, a plethora of expression data about microglia in health and disease has lately become available (among others Gosselin et al., 2017; Friedman et al., 2018; Saunders et al., 2018; Hammond et al., 2019). However, the specific tasks of differentially regulated genes and their interactions in microglia remain largely unknown, highlighting the importance of understanding the **Cell Biology of Microglia**. Thus, this “Research Topic” includes six research articles and one review about intracellular mechanisms that drive microglial functions under physiological and pathological conditions.

Three of the research articles published in this Research Topic deal with microglial responses to extracellular cues and their behavior in the CNS. Lisi et al. confirm the expression of the Nerve Growth Factor (NGF) receptors, namely TrkA and p75NTR, in two human microglial cell lines. In addition, they determine the effect of NGF and its painless human recombinant mutated analog (hNGFp) on microglia activation. The authors also discuss how hNGFp appears to induce a better-defined anti-inflammatory profile compared to wild type NGF, which might improve NGF-treatment in some neuropathies. Corraliza-Gomez et al. analyze in detail the role of Apolipoprotein D (ApoD), a lipid-binding protein of the Lipocalin family, in microglial responses. ApoD, which plays an important role in CNS maintenance, increases its expression upon aging, damage or neurodegeneration. Here, the authors demonstrate that extracellular ApoD can be sensed by microglia, affecting their number and functions, and triggers cytokine signatures that are stimuli- and sex-dependent. They propose ApoD as a “priming or immune training factor”, whose exposure during life might modulate acute microglial damage responses later on. Similarly, Fernández-Arjona et al. also explore immune priming in the brain upon exposure to an immunological challenge early in life. They studied its persistence over time and the relevance of toll-like receptors (TLRs) in this process. Using a mouse model and a double stimulus paradigm design, the authors claim that such immune priming events in the brain are largely dependent on TLR4 in their experimental settings.

One of the most widely used ligands of TLR4 to induce inflammation is the lipopolysaccharide (LPS) from bacterial origin. Martín-Sierra et al. describe how LPS-stimulated microglia can induce ganglion cell death by an NO-dependent mechanism in organotypic cultures of quail embryo retina. Since phagocytic contacts between microglia and caspase-3-positive ganglion cells are increased upon LPS-treatment, the authors discuss the possibility of a cell death mechanism mediated by microglial engulfment. Working as well with retinal microglia, the following article by Wang et al. describes a new and efficient protocol to isolate primary retinal microglia from adult human post-mortem eyes. The characterization in terms of morphology, markers and response to LPS converts this method into a great tool to study the pathogenesis of inflammatory ocular diseases.

Based on the crosstalk between microglia and other glial cells, the work of Guo et al. investigate the dynamic interactions between the translocator protein (TSPO) as neuroinflammatory marker, microglia and astrocytes in a mouse model of depression. These glial interactions are examined *in vivo* in different hippocampal subregions by a combination of positron emission tomography (PET) technology with TSPO ligands, double-immunofluorescence and cytokine analysis.

Finally, a review article by Gabrielli et al. summarizes the current knowledge about extracellular vesicles released by microglia, including their composition, isolation and purification methods. This comprehensive review also discusses their heterogeneous properties and multifaceted effects as novel intercellular communication system.

In conclusion, this Research Topic contains several important articles for Frontiers in Cellular Neuroscience readers interested in the latest findings in microglial cell biology.

## Author contributions

MRS and VEN wrote the manuscript. All authors revised, contributed to the article, and approved the submitted version.
